# Ungewöhnliche Ursache eines pulmonalen Infektes: Es ist nicht immer eine Tuberkulose

**DOI:** 10.1007/s00117-025-01426-0

**Published:** 2025-03-05

**Authors:** Christopher Kloth, Thomas Breining, Annika Beck, Camilla Westerwinter, Giuseppe Zenatti, Meinrad Beer, Daniel Vogele

**Affiliations:** 1 Klinik für Diagnostische und Interventionelle Radiologie, Universitätsklinikum Ulm, Albert-Einstein-Allee 23, 89081 Ulm, Deutschland; 2 Institut für Pathologie, Universitätsklinikum Ulm, Ulm, Deutschland; 3 Klinik für Innere Medizin III, Universitätsklinikum Ulm, Ulm, Deutschland

## Anamnese

Wir berichten über einen 31-jährigen Patienten, der sich notfallmäßig in der zentralen Notaufnahme unseres Klinikums mit respiratorischer Beeinträchtigung und blutigem Sputum vorgestellt hat. Klinisch zeigten sich Abgeschlagenheit, Hämoptysen seit 2 Tagen sowie intermittierendes Fieber. Die Sauerstoffsättigung unter Raumluft lag bei 88 %.

Anamnestisch hatte der Patient unmittelbar Kontakt zu einem Angehörigen mit offener Lungentuberkulose. Aufgrund des Kontaktes erfolgte die Verlegung und Aufnahme auf die Infektionsstation unserer Klinik. Bei der weiteren Anamnese berichtete der Patient über eine abgelaufene Hepatitis-C-Infektion und einen i.v.-Drogenabusus. Seit einem Monat vor der aktuellen Vorstellung nahm der Patient an einem Substitutionsprogramm mit Methadon im ambulanten Bereich teil (L-Polamidon, Beikonsum von Kokain und Heroin).

Laborchemisch zeigte sich eine chronische Hepatitis-C-Infektion sowie deutlich erhöhte Infektparameter (CRP 130,0 mg/l [< 5,0 mg/l], Leukozyten 16,6 Giga/l [4,4–11,3 Giga/l]). Direkt bei Aufnahme wurde mit einer empirischen Antibiotika-Therapie mit Piperacillin/Tazobactam und Vancomycin begonnen.

## Radiologische Diagnostik

Zur weiteren Diagnostik wurde eine Bildgebung mittels nativer Computertomographie (CT) des Thorax zu Eruierung eines pulmonalen Infektgeschehens mit Verdacht auf eine mögliche floride Tuberkulose veranlasst. Bei dem anamnestisch bekannten Drogenabusus stand als Differenzialdiagnose ein atypischer pulmonaler Infekt, z. B. pilzbedingt zur Diskussion.

**Abb. 1:**
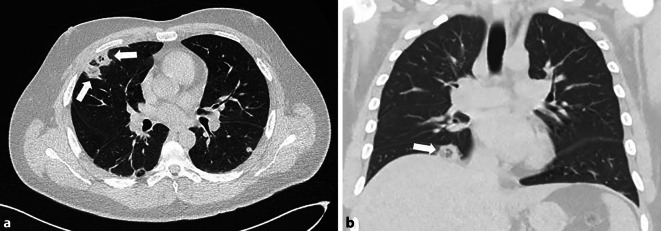
Native Computertomographie (CT) des Thorax im Lungenfenster mit axialen (**a**) und koronaren (**b**) Rekonstruktionen. Überwiegend peripher lokalisiert lassen sich im Mittellappen (**a**) und am Zwerchfell rechts im basalen Unterlappen (**b**) unscharfe Verdichtungen mit Lufteinschlüssen und umgebender Milchglastrübung abgrenzen (*Pfeile*)

In der durchgeführten CT (Abb. 1) zeigten sich stark basal betonte, subpleurale, bipulmonale, rundliche Infiltrate mit zentralen Lufteinschlüssen und umgebendem Milchglas. Tuberkuloseverdächtige Kavernen waren nicht abgrenzbar.

## Wie lautet Ihre Diagnose?

## Definition

Lungenrundherde sind gemäß ihrer Anordnung in ein perilymphatisches Verteilungsmuster, ein zentrilobuläres Verteilungsmuster (endobronchiale Streuung) und ein zufälliges Verteilungsmuster (hämatogene Aussaat) einzuordnen [1]. Bei einschmelzenden Lungenrundherden mit zufälligem/hämatogenem Verteilungsmuster sind als Differenzialdiagnose septische pulmonale Embolien zu berücksichtigen. Es handelt sich dabei um zumeist bilaterale Lungenrundherde unter Betonung der Lungenunterlappen. Die basalen Lungenabschnitte sind aufgrund des Euler-Liljestrand-Mechanismus vermehrt betroffen. Die Lungenperipherie wiederum ist aufgrund der sich hier aufzweigenden und immer kleiner werdenden Gefäße Hauptlokalisationsort [4].

Die Rundherde sind meist scharf abgrenzbar und können rasch entstehende Einschmelzungen aufweisen. Die Größe und der Grad der Einschmelzung kann je nach dem unmittelbaren Entstehungsalter variieren. Auf bildgebend typische Zeichen wie eine angiotope Lage mit etwaigem „feeding vessel sign“ ist zu achten [2, 5]. Das Vorliegen dieses Zeichens wird in der Literatur mit 70–100 % beschrieben. Weiteres typisches Erscheinungsbild ist ein Halo-Saum um die Herde, wobei hier eine zugrunde liegende Einblutung bzw. Infarktareal angenommen wird [4]. Insgesamt kann das Erscheinungsbild je nach zugrunde liegendem Erreger variieren. Für grampositive Erreger ist ein größerer Durchmesser der Einzelherde beschrieben [4]. Ein begleitender Pleuraerguss kann in bis zu 70 % der Fälle vorliegen.

Als zugrundliegender Entzündungsfokus kommen vielfältige Ursachen in Frage wie eine infektiöse Endokarditis, tiefe Venenthrombosen, dentaler Fokus, Osteomyelitis, Hautinfektionen oder venöse periphere Zugänge [4]. Als häufigste Ursache wird in der Literatur ein i.v.-Drogenabusus beschrieben, wie auch im vorliegenden Fall [5].

*Staphylococcus aureus* ist der häufigste Keim bei i.v.-Drogenabusus im Rahmen von Hautinfektionen und katheterassoziierten Streuherden [3]. Auch im vorliegenden Fall war eine *S.-aureus*-Bakteriämie in der Blutkultur nachweisbar. Die CT ist oftmals einem auffälligen Blutkulturbefund zeitlich voraus, bevor hier ein Keimnachweis gelingt.

Die Symptome sind zumeist unspezifisch mit etwaiger Luftnot, Husten, respiratorischen Beschwerden und Fieber [3]. Auf begleitende Symptome des ursächlichen Primärfokus ist zu achten.

Bildgebend ist die CT das Mittel der ersten Wahl. Auch bei Röntgenaufnahmen des Thorax mit entsprechendem klinischem Hintergrund ist auf etwaige Lungenrundherde zu achten. Im Falle einer verzögerten Diagnosestellung kann es zu einer Sepsis oder zur weiteren hämatogenen Streuung kommen.

Im vorliegenden Fall war die initiale CT-Bildgebung der Lunge der ausschlaggebende Hinweis, um eine weiterführende detaillierte Fokussuche zu initiieren. Eine Diagnosesicherung mittels Blutkulturen kann, muss aber nicht wegweisend sein, je nachdem, wie lange die Primärinfektion besteht. Im vorliegenden Fall war das Ergebnis der Blutkultur positiv.

Differenzialdiagnostisch sind bei einschmelzenden Lungenrundherden mit zufälligem/hämatogenem Muster in erster Linie Metastasen, Abszesse, Rheumaknoten oder eine etwaige Granulomatose mit Polyangiitis abzuwägen. Therapeutisch ist eine zielgerichtete Antibiose sowie die Therapie des Primärfokus Mittel der Wahl.

## Therapie und Verlauf

In der CT zeigte sich kein typisches Erscheinungsbild für eine primäre Tuberkulose (Abb. 1). Diese konnte auch anhand der negativen, mikroskopischen Untersuchungen von Urin, Stuhl und Sputum mehrfach ausgeschlossen werden. Zudem war der Tuberkulose-Bluttest negativ.

**Diagnose:** Hämatogene septische Streuherde.

**Abb. 2:**
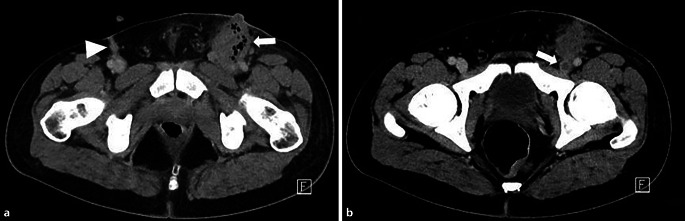
Kontrastmittelgestützte Computertomographie (CT) des Beckens im Weichteilfenster. **a** Links inguinal lässt sich ein Abszess (*Pfeil*) ventral der Gefäße mit Ausbreitung bis zur Kutis abgrenzen. Auf der Gegenseite finden sich rechts inguinal ebenfalls ventral der Gefäße narbig imponierende Veränderungen (*Pfeilspitze*). **b** Zusätzlich ist in der linken V. femoralis communis eine Thrombose abzugrenzen (*Pfeil*)

Auf dem Boden der Verdachtsdiagnose möglicher septischer Streuherde in der CT erfolgte eine nochmalig gezielte intensive Fokussuche. Hierbei ergab sich ein ca. 4 cm großer Abszess in der linken Leiste (Abb. 2). Dieser Abszess war am ehesten auf dem Boden des vorangegangenen i.v.-Drogenkonsums in der Leiste entstanden.

Über den Verlauf zeigte sich ferner eine venöse Thrombose in der Leiste (V. feoralis communis). Weitere Streuherde in der Muskulatur des Beckens sowie an den Beinen zeigten sich in einer PET/CT-Untersuchung im Verlauf.

**Abb. 3:**
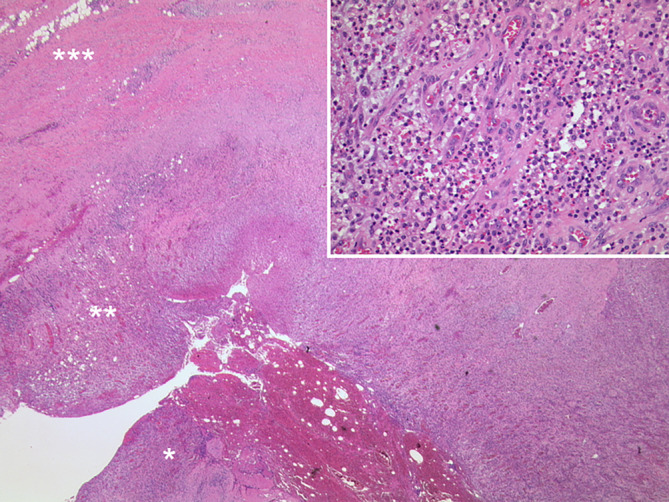
Histopathologie des Präparats nach chirurgischer Exzision in der linken Leiste. Links unten befindet sich die Abszesshöhle (*Stern*) mit nekrotischem Material, Blut und neutrophilen Granulozyten. Diese ist von einer Zone aus Granulationsgewebe (**) umgeben, die von einer Fibrose (***) umrandet ist. (HE-Färbung, Originalvergrößerung 12,5:1. Vergrößerung: Granulationsgewebe mit einem entzündlichen Infiltrat reich an neutrophilen Granulozyten. HE-Färbung, Originalvergrößerung 200:1)

Therapeutisch erfolgte die gefäßchirurgische Exzision des Abszesses und die Revaskularisation der V. femoralis communis mittels Venenpatch aus der V. saphena magna. In der histopathologischen Aufarbeitung zeigte sich das Bild einer Abszedierung (Abb. 3). Der Patient entließ sich 8 Tage nach dem erfolgten gefäßchirurgischen Eingriff selbst gegen ärztlichen Rat.

## Fazit für die Praxis

- Bei vorliegenden Risikofaktoren (z. B. infektiöse Endokarditis, Zahnfokus, Hautinfektionen, venöse periphere Zugänge oder i.v.-Drogenabusus) ist bei einschmelzenden Lungenrundherden an septische Streuherde zu denken.
- Lungenrundherde sind gemäß ihrer Anordnung in ein perilymphatisches, zentrilobuläres oder zufälliges Verteilungsmuster mit den dazugehörigen Differenzialdiagnosen einzuordnen.
- Wichtig ist neben der objektiven Bildanalyse der Computertomographie (CT) auch die Korrelation mit der klinischen Symptomatik und der Anamnese des Patienten.
